# Later-Life Health Disparities in Nepal: Intersection of Gender and Socioeconomic Status

**DOI:** 10.1093/geroni/igaa057.1666

**Published:** 2020-12-16

**Authors:** Tirth Bhatta, Neema Langa, Nirmala Lekhak, Denise Burnette

**Affiliations:** 1 University of Las Vegas Nevada, Las Vegas, Nevada, United States; 2 UNLV, Las Vegas, Nevada, United States; 3 University of Nevada, Henderson, Nevada, United States; 4 VCU, Richmond, Virginia, United States

## Abstract

This study focuses on Nepal, a country still undergoing capitalist expansion, to examine intersectional effects of fundamental causes on later life health outcomes. Sandwiched between the republic of China and India, Nepal still has remnants of pre-capitalist social and economic formations. Despite growing focus on independent effects of SES and gender on health, the intersectional influences of such fundamental causes on later life health in Nepal has, however, been largely unexplored. Drawing from the World Health Survey (WHS) survey data (n=2,250 aged 50 years and older), we rely on negative binomial regression models to examine whether the effect of education and household wealth on chronic diseases and functional limitations differs between men and women. Findings indicate intersectional effects of gender, wealth, and education on health. Women do not incur health benefits from education and wealth. Statistically significant negative effect of education on functional limitations (OR=0.87, p<0.01) was documented only for men. Contrary to our theoretical expectations, we observed significantly higher count of chronic diseases among women (OR=1.13, p<0.01) with higher levels of education relative to lower educated women. Similarly, men in higher wealth quintiles reported significantly higher count of chronic diseases (OR=1.05, p<0.01) than their counterparts in lower wealth quintiles. Our study paves a way for future research on a range of structural mechanisms such as gendered labor market, patriarchal cultural expectations, and inequities in health care that could mediate intersectional effects of gender and education on later life health disparities in the Global South.

